# Process and Methodological Considerations for Observational Analyses of Vector Control Interventions in Sub-Saharan Africa Using Routine Malaria Data

**DOI:** 10.4269/ajtmh.22-0757

**Published:** 2023-08-21

**Authors:** Sarah M. Burnett, Kelly M. Davis, Gudissa Assefa, Christelle Gogue, Levi D. Hinneh, Megan Littrell, Julia Mwesigwa, Okefu O. Okoko, Saraha Rabeherisoa, Musa Sillah-Kanu, William Sheahan, Hannah C. Slater, Perpetua Uhomoibhi, Frederick Yamba, Kelley Ambrose, Kathryn Stillman

**Affiliations:** ^1^ U.S. President’s Malaria Initiative (PMI) VectorLink Project, PATH, Washington, District of Columbia;; ^2^ National Malaria Elimination Programme, Addis Ababa, Ethiopia;; ^3^ New Nets Project, PATH, Seattle, Washington;; ^4^ National Malaria Control Programme, Monrovia, Liberia;; ^5^ PATH, Seattle, Washington;; ^6^ New Nets Project, PATH, Kampala, Uganda;; ^7^ National Malaria Elimination Programme, Abuja, Nigeria;; ^8^ Programme National de Lutte Contre le Paludisme, Antananarivo, Madagascar;; ^9^ National Malaria Control Program, Freetown, Sierra Leone;; ^10^ President’s Malaria Initiative (PMI) VectorLink Project, Abt Associates, Rockville, Maryland

## Abstract

Progress in malaria control has stalled in recent years. With growing resistance to existing malaria vector control insecticides and the introduction of new vector control products, national malaria control programs (NMCPs) increasingly need to make data-driven, subnational decisions to inform vector control deployment. As NMCPs are increasingly conducting subnational stratification of malaria control interventions, including malaria vector control, country-specific frameworks and platforms are increasingly needed to guide data use for vector control deployment. Integration of routine health systems data, entomological data, and vector control program data in observational longitudinal analyses offers an opportunity for NMCPs and research institutions to conduct evaluations of existing and novel vector control interventions. Drawing on the experience of implementing 22 vector control evaluations across 14 countries in sub-Saharan Africa, as well as published and gray literature on vector control impact evaluations using routine health information system data, this article provides practical guidance on the design of these evaluations, makes recommendations for key variables and data sources, and proposes methods to address challenges in data quality. Key recommendations include appropriate parameterization of impact and coverage indicators, incorporating explanatory covariates and contextual factors from multiple sources (including rapid diagnostic testing stockouts; insecticide susceptibility; vector density measures; vector control coverage, use, and durability; climate and other malaria and non-malaria health programs), and assessing data quality before the evaluation through either on-the-ground or remote data quality assessments. These recommendations may increase the frequency, rigor, and utilization of routine data sources to inform national program decision-making for vector control.

## INTRODUCTION

Malaria vector control interventions have contributed to decreases in malaria transmission since the early 2000s.^1^ Although global malaria case incidence decreased by 27% from 81 to 59 cases per 1,000 population at risk from 2000 to 2015, progress has stalled in recent years, with malaria incidence holding at 59 cases per 1,000 population at risk in 2020.^2^ Some potential reasons for this lack of progress include reduced funding for malaria control, low political will or engagement, plateaus in vector control coverage, remote areas not being reached by vector control interventions, and mosquito populations becoming increasingly resistant to the insecticides used in existing vector control tools.^3^^–^^5^ However, new vector control tools are available to address these challenges. Since 2017, the WHO has prequalified two new active ingredients for indoor residual spraying (IRS) products—pirimiphos-methyl and clothianidin—and three new active ingredients that are combined with pyrethroids in insecticide-treated net (ITN) products—piperonyl butoxide (PBO), chlorfenapyr, and pyriproxyfen.^6^ But these new tools often come at a greater financial cost.^7^^–^^9^

With recent changes in WHO policy to accelerate product evaluation and rapid scaling of new interventions, not all new vector control products are required to undergo cluster randomized controlled trials (CRCTs) with epidemiological outcomes to receive WHO prequalification.^10^ The goal of CRCTs is to assess the efficacy of the vector control product under ideal conditions, which may not be representative of routine implementation. Cluster randomized controlled trials, conducted primarily before the introduction and scale-up of a new tool, provide the highest quality of evidence of effectiveness. However, primarily because of cost, randomized controlled trials (RCTs) are often completed in only a few countries.^11^^–^^14^ When the deployment of new vector control tools is considered, national malaria control programs (NMCPs) need to be able to make data-driven, cost-effective decisions to make more efficient use of limited resources and maximize impact. Ministries of health (MOHs) and NMCPs are increasingly supporting subnational stratification and targeted implementation of selected vector control interventions, with data sources and strategies varying across countries.^15^^–^^18^ However, there is often limited evidence regarding the impact of these new tools, or combinations of tools, within specific country contexts to support product choice and targeting decisions. Quasi-experimental designs are often used to evaluate the impact of vector control tools during program scale-up by assessing any observed changes in trends and testing association with predictor variables.^19^^,^^20^

The increased availability and quality of routine malaria case data from health management information systems (HMIS) has greatly improved, with health facility and in some cases community- or individual-level data available within national databases, such as District Health Information Software 2 (DHIS2), a web-based open-source software that is used as the HMIS in 73 countries.^21^^–^^24^ There have also been recent improvements in data collection and management systems for vector control, with increased availability of entomological and vector control program data from NMCPs, research institutions, and implementing partners. Although further work is needed to strengthen vector control data systems in many countries, increasingly MOHs and NMCPs are integrating entomology and vector control data into their DHIS2 systems. This is often implemented through (1) the addition of custom-built programs or (2) the use and adaptation of entomology and vector control DHIS2 modules developed by WHO or VectorLink Collect DHIS2 modules developed by the U.S. President’s Malaria Initiative (PMI) VectorLink Project.^25^ These efforts have improved the availability of routine data to help address important malaria control questions.

Through well-designed, quasi-experimental evaluations using routine epidemiological, entomological, and programmatic (i.e., campaign data) data sources, NMCPs can assess the impact of vector control interventions in their countries and inform national policy, strategy, and implementation. Leveraging these routine data sources can allow NMCPs to conduct impact evaluations at a lower cost, at a more granular level, and with greater breadth across the country than through RCTs or other primary data collection methods. Although general guidance exists for using routine data to evaluate malaria interventions, there is insufficient practical and specific guidance on the design and implementation of vector control evaluations using routine data.^22^^,^^26^ Informed by a narrative review of published literature and project experience in conducting vector control evaluations, this article provides considerations for (1) selecting study objectives and designs, including follow-up periods; (2) defining outcome and exposure variables; (3) identifying covariates and contextual factors, their calculations, and data sources to strengthen the analysis; (4) addressing common data quality challenges; and (5) using evaluation findings to inform national policy, strategy, and program implementation. This practical guidance can increase the frequency, rigor, and utilization of routine data to support national and subnational vector control decision-making as well as help standardize analysis approaches to allow for cross-country comparisons.

## MATERIALS AND METHODS

**Figure 1.**
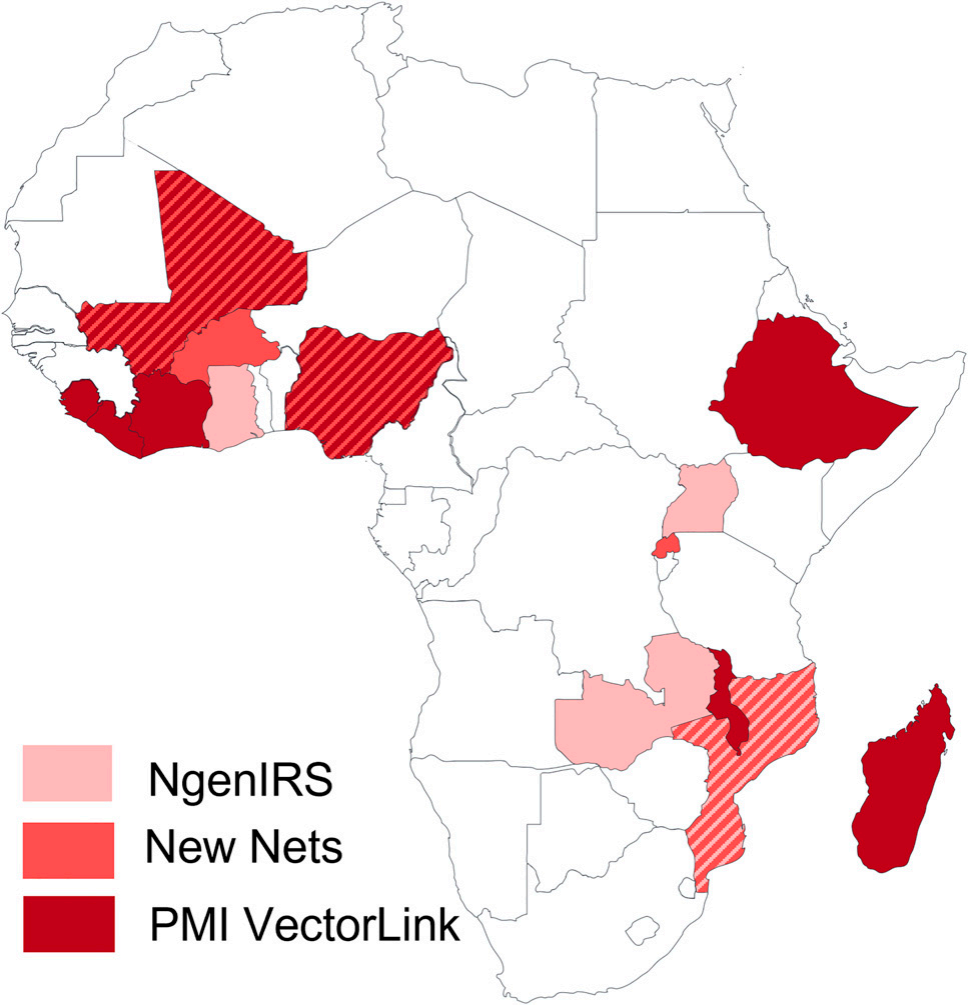
Map of countries with vector control evaluations under NgenIRS, the New Nets Project, and PMI VectorLink. NgenIRS = Next Generation IRS; PMI = U.S. President’s Malaria Initiative.

From 2016 to 2022, 22 retrospective longitudinal studies assessing the impact of vector control interventions on malaria case incidence using routine data across 14 countries in sub-Saharan Africa have been conducted or are still underway by the Next Generation IRS (NgenIRS) project, the New Nets Project, and the PMI VectorLink Project, in partnership with NMCPs and local research institutions (see Figure 1 and Table 1).^27^^–^^35^ In this analysis, the lessons learned and methodologies from these evaluations were collated and reviewed through discussions with eight program staff members, including details on how various epidemiological, entomological, and vector control data were used to assess the impact of interventions on malaria disease burden. To supplement information from our experience conducting evaluations, we searched PubMed for retrospective longitudinal studies assessing the impact of IRS and ITNs, focusing on evaluations that use outcome indicators from routinely collected data, including malaria cases, malaria case incidence, malaria hospitalization or death, test positivity rate, or low birth weight. Through this process, 11 vector control impact evaluations that used routine health systems data were selected, as they described key characteristics of the evaluation for each of the areas of consideration in this manuscript, including the evaluation questions, time period of analysis, evaluation design and analysis, unit of analysis, outcome, exposure, covariates included in the model, contextual variables, and data quality checks conducted (see Supplemental Table 1).

**Table 1:** Vector control evaluations using routine HMIS data conducted or planned by NgenIRS, the New Nets Project, and PMI VL*

Country	Project	Evaluation Question(s)	Time Period	Design and Analysis	Unit of Analysis	Outcome	Exposure	Covariates	Contextual Variables	Data Quality Checks
Burkina Faso	PMI VL	(1) What is the impact of the 2018 and 2020 IRS campaigns on malaria case incidence on top of nationwide SMC and ITN campaigns? (2) Are there differential impacts by region?	July 2016–Apr 2021; 25 months pre-2018 IRS, 11 months post-2018 pre-2020, and 10 months post-2020 IRS	Interrupted time series analysis using a log-linear regression model; control group was two districts that did not receive IRS	Health facility; 65 health facilities in four districts	Laboratory-confirmed malaria cases per 100 non- OPD visits	IRS status (binary)	Rainfall (lagged 2 months), EVI (lagged 1 month), regional high-transmission season	IRS spray coverage (structures sprayed/found), IRS population coverage (population protected/total population)	Data from June 2019 to May 2020 were removed owing to HMIS reporting issues; checked completeness and for outliers
Cote d’Ivoire*	PMI VL	What is the impact of the 2020 and 2021 IRS campaigns?	Sept 2018–Apr 2022; 24 months pre-2020 IRS, 11 months between 2020 and 2021 IRS, and 8 months post-2021 IRS	Interrupted time series analysis using a log-linear regression model; control group was two districts that did not receive IRS	Health facility; 88 health facilities in four districts	Confirmed malaria cases per 1,000 population	IRS status (binary)	RDT stockouts, rainfall (lagged 2 months), temperature (lagged 2 months) EVI (lagged 2 months), high transmission season, proportion of cases reported by CHWs, non-malaria outpatient visits.	IRS spray coverage (structures sprayed/found), insecticide resistance, IRS residual efficacy	Data were collected from registries owing to District Health Information Software 2 quality concerns; data from September 2019 to August 2020 removed owing to significant RDT stockouts
Ethiopia	PMI VL	What is the impact of the 2015–2019 IRS campaigns?	May 2015–Apr 2019; 48 months post-IRS	Dose-response approach with a negative binomial distribution and a log population offset	District; 34 districts in two regions	Confirmed malaria cases per 1,000 population	IRS population coverage (% of population protected by IRS)	Rainfall (lagged 2 months), NDVI (lagged 1 month), terms for month and spray year	Human biting rate, IRS residual efficacy, ITN distribution	Excluded district-months if IRS population coverage was low (< 50%), were missing malaria case data, or were not sprayed that year
Ethiopia*	PMI VL	Are PBO ITNs as effective as IRS plus standard pyrethroid ITNs?	July 2019–June 2024; 24 months pre- and 36 months post-intervention	Cluster-randomized noninferiority trial	Health facility; 57 health posts in five districts	Confirmed malaria cases per 100 non-OPD visits†	Intervention status (binary)	ITN distribution, ITN usage, IRS coverage, rainfall, EVI, elevation	Human biting rate, species composition, sporozoite rate, insecticide residual efficacy, insecticide resistance	Data quality checks to determine the percentage of facilities with available data
Liberia*	PMI VL	What is the impact of IG2 ITNs?	Aug 2019–June 2022; 24 months pre- and 22 months post-intervention	Interrupted time series analysis using a negative binomial model with no control group	Separate analyses at the health facility and district levels; 500+ facilities in 15 districts	Confirmed malaria cases per 1,000 population	ITN status (binary)	ITN distribution, rainfall, NDVI, RDT stockouts, ACT stockouts	Vector density, species composition, EIR, insecticide susceptibility	Data quality checks to determine the percentage of facilities with available data
Madagascar	PMI VL	(1) What is the impact of IRS? (2) What is the additional impact, if any, of consecutive years of IRS? (3) What is the impact of IRS coverage >85%?	July 2016–June 2021; 60 months, with pre/post period varying by location	Counterfactual scenarios of no IRS vs. IRS generated using multilevel, negative binomial, generalized linear mixed-effects model	Health facility; 2,524 facilities across all districts	Confirmed malaria cases per 1,000 population	IRS status—set of binary variables indicating months post-campaign (1–6 or 7–12 months) and number of consecutive IRS years; IRS coverage (sprayed out of found structures)	ITN survivorship, mass drug administration (binary), rainfall (lags varied by part of country), EVI	None	Facilities missing geocoordinates, population, and private health facilities and hospitals were excluded; health facility months missing RDT-confirmed case data were excluded from initial effect estimates; cases were imputed using the model, and these facility months were included in the final model
Malawi*	PMI VL	(1) Is IRS as effective as dual-active ingredient (AI) and PBO ITNs? (2) Are dual-AI ITNs as effective as PBO ITNs? (3) What are the relative impacts of two dual-AI ITNs (IG2 and Royal Guard)?	Jan 2018–May 2024; ∼46–53 months pre- and 24–30 months post-interventions; varies by month of implementation	Interrupted time series analysis with comparison group using a multilevel, negative binomial, generalized linear mixed-effects model	Health facility; 998 facilities in all 29 districts	Confirmed malaria cases per 1,000 population	Intervention status (IRS, Royal Guard ITN, IG2 ITN, PBO ITN, mixed ITNs)	Age (< 5, 5+) years, rainfall (lagged), EVI (lagged), elevation, IRS spray coverage, ITN population coverage, non-malaria outpatient attendance, % treated at community level	Species composition, indoor resting density, human biting rate, EIR, insecticide resistance, parity rate	Analysis restricted to facilities with 100% complete reporting; imputation methods will be explored if <60% of facilities do not meet this criterion
Mali	PMI VL	What is the impact of the 2017–2019 IRS campaigns?	June 2016–May 2019; 12 months pre- and 24 months post-campaign; separate analysis for annual transmission and high transmission season only	Difference-in-difference using a negative binomial model; control group was those facilities that did not receive IRS	Health facility; 47 facilities (annual transmission) and 63 facilities (high-transmission season only)	Confirmed malaria cases per 1,000 population	IRS status (binary)	Rainfall (lagged 1 month), NDVI (lagged 2 months)	Human biting rate, IRS residual efficacy, insecticide susceptibility, < 5 malaria prevalence, health-seeking behavior, ITN ownership	Analysis restricted to facilities with 100% complete reporting during the study period
Mali*	PMI VL	What is the impact of IG2 ITNs?	Aug 2018–July 2022; 24 months pre- and 24 months post-campaign	Interrupted time series analysis with comparison group using a multilevel, negative binomial, generalized linear mixed-effects model	Health facility; 240 facilities in 10 districts	Confirmed malaria cases per 1,000 population	ITN status (binary)	ITN distribution, rainfall, NDVI, SMC coverage, intermittent preventive therapy in pregnancy coverage, RDT and ACT stockouts	ITN ownership and use, vector composition, vector density, vector behavior, insecticide susceptibility	Data completeness checks will be done for each facility; imputation may be done for facilities missing 1 month of data during study period if completeness falls below 60%
Nigeria*	PMI VL	What is the impact of PBO ITNs?	Dec 2017–Nov 2021; 24 months pre- and 24 months post-campaign	Interrupted time series analysis using a negative binomial model with control group	Ward; 216 wards in one state	Confirmed malaria cases per 1,000 population	ITN status (binary)	Rainfall (lagged), EVI (lagged), RDT stockouts, ITN use	Vector density, vector behavior, vector composition, parity rates, EIR, insecticide susceptibility	Data completeness checks done for each ward and trends by completeness threshold reported
Nigeria*	PMI VL	What are the relative impacts of the IG2 and PBO ITNs?	Nov 2018–Oct 2023; 36 months pre- and 24 months post-campaign	Interrupted time series analysis using a negative binomial model with comparison group	Facility; 1,709 facilities in two states	Confirmed malaria cases per 1,000 population	ITN status (binary); ITN type (binary)	Rainfall (lagged), EVI (lagged), RDT stockouts, ITN use	Vector density, vector behavior, vector composition, parity rates, EIR, insecticide susceptibility	Data completeness checks done for each ward and trends by completeness threshold reported
Sierra Leone*	PMI VL	Is IRS plus PBO ITNs more effective than PBO ITNs only?	May 2018–Apr 2023; 36 months pre- and 24 months post-campaign	Interrupted time series analysis using a negative binomial model with comparison group	Health facilities in four districts	Confirmed malaria cases per 1,000 population†	Intervention status (PBO ITN vs. IRS + PBO ITN)	Non-malaria outpatient visits, ITN coverage, ANC and EPI ITN coverage, rainfall, EVI, RDT stockouts	Entomological indicators (i.e., vector density, vector behavior, vector composition, insecticide resistance)	Data completeness checks will be done for each facility
Ghana^27^	NgenIRS	(1) What is the epidemiological impact of IRS? (2) What is the impact of withdrawing IRS?	Jan 2015–Dec 2017; 6-month follow-up after each round of IRS	Difference-in-difference approach used to assess implementation and withdrawal of IRS	District; 35 districts in three regions	RDT-confirmed malaria cases per 10,000 person-months	IRS status (binary)	None reported	Temperature	Data completeness was reported for each region
Mali^28^	NgenIRS	(1) What is the impact of implementing IRS in districts that did not receive IRS previously? (2) What is the impact of suspending IRS operations?	Jan 2016–Mar 2018; 6-month follow-up after each round of IRS	Difference-in-difference approach used to assess both implementation and withdrawal of IRS	Health facility; 314 facilities in two regions	RDT-confirmed malaria cases (< 5 years) per 10,000 child-months	IRS status (binary)	None reported	SMC campaign data, % of suspected cases receiving diagnosis, % of confirmed cases treated with ACT, RDT stockouts	Data completeness was reported for each district
Mali^29^	NgenIRS	What is the impact of IRS and SMC, either individually or in combination, on malaria case incidence?	June 2014–Mar 2015; 6-months post-IRS and first round of SMC	Post-only comparison using a negative binomial regression model to compare intervention districts to districts receiving neither IRS nor SMC	District; six districts in one region	RDT-confirmed malaria cases per 10,000 person-months, by all ages and < 5 years	Intervention status (IRS, SMC, IRS + SMC, none)	None reported	None reported	Censored facility-months with no data from the analysis
Mali^30^	NgenIRS	(1) What is the impact of non-pyrethroid IRS? (2) What is the cost-effectiveness of IRS? (3) How would the impact change if the campaign start date changed? (4) What is the impact of IRS withdrawal?	Jan 2012–Aug 2015; 6 months post-IRS each year	Post-only comparison between IRS and non-IRS districts	District; seven districts in one region	RDT-confirmed malaria cases per 10,000 person-months	IRS status (binary)	None reported	ITN access and use, SMC coverage, RDT stockout, entomology surveillance, rainfall	Removed years when a district received SMC to independently evaluate IRS; district-months with no data excluded
Mozambique^31^^,^^32^	NgenIRS	(1) What is the benefit of IRS plus ITNs? (2) What is the cost-effectiveness of IRS plus ITNs?	Nov 2016–Oct 2018; 10 months post-first IRS campaign, 13 months post-second IRS, 17 months post-ITNs	Negative binomial regression model using generalized estimating equations to compare IRS and non-IRS clusters	Health facility	Confirmed malaria cases per 10,000 person-months†	IRS status (binary)	Net usage, net integrity and washes, and ecological, sociological, and economic variables	Malaria prevalence, vector density, human biting rate, sporozoite rate, insecticide susceptibility, EIR	None reported
Zambia^33^	NgenIRS	(1) What is the epidemiological effectiveness of introducing Actellic 300CS for IRS in Zambia? (2) What is the additional/combined effect of incorporating the mSpray/REVEAL tool?	Dec 2013–June 2017; 7 months after each IRS campaign over 3 years	Random effects Poisson model used to assess the relationship between IRS exposure and incidence; comparison group was facility catchment areas not receiving IRS	Facility	Confirmed malaria case incidence per 1,000 population	IRS and mSpray status (no IRS, IRS only, IRS w/ mSpray)	EVI (lagged 1 month), precipitation (lagged 2 months), province and year fixed effects, total number of tests performed	IRS coverage	Facilities were dropped if they could not be linked for geographical and environmental data; facilities only included if data were available for at least 3 of the 7 months
Burkina Faso, Rwanda, Mozambique, Nigeria^34^	New Nets	(1) What is the impact of IG2, Royal Guard, and PBO ITNs compared with standard ITNs?	Varies by country 2018–2022; 12 months pre- and 24 months post-campaign	Pre/post comparison using a difference-in-difference approach to compare impact of dual-AI ITNs to standard ITNs	Health facility; district; LGA (varies by country)	Confirmed malaria case incidence per 10,000 person-months	ITN status by type	Appropriate covariates will be identified for each country	ITN survivorship, attrition, and physical integrity; human behavior	Data quality assessments being conducted in some country to assess quality and completeness of malaria surveillance data

ACT = artemisinin combination therapy; ANC = antenatal coverage; EIR = entomological inoculation rate; EPI = Expanded Programme on Immunization; EVI = enhanced vegetation index; HMIS = health management information system; IG2 = interceptor G2; IRS = indoor residual spraying; ITN = insecticide-treated net; LGA = local government area; NDVI = normalized difference vegetation index; NgenIRS = Next Generation IRS; OPD = outpatient department; PBO = piperonyl butoxide; PMI = U.S. President’s Malaria Initiative; RDT = rapid diagnostic test; SMC = seasonal malaria chemoprevention; VL = VectorLink.

* Evaluations are ongoing, and details are subject to change.

† In addition to the listed epidemiological outcomes from routine HMIS data, these studies also include entomological outcomes that are prospectively collected from sentinel sites.

## RESULTS AND DISCUSSION

### Selecting study objectives and designs.

#### Objectives of vector control evaluations.

Vector control impact evaluations typically assess the impact of vector control tools on clinical outcomes such as malaria cases or malaria case incidence.^27^^–^^34^^,^^36^^–^^58^ Beyond assessing the impact of vector control interventions, vector control and NMCP leadership should identify the most relevant policy questions, including any cost-benefit analyses, that can be answered with the available data. The evaluations should be done as often as needed to effectively inform national policy and decision-making. In addition to using routine malaria case data collected from health facilities to assess overall impact, these data could be used to address secondary questions such as (1) heterogeneity in impact between different regions,^59^ (2) withdrawal of interventions,^27^^,^^30^^,^^51^^,^^55^ (3) level of coverage of the intervention,^38^ (4) duration of impact after a single campaign,^55^ (5) effects of multiple campaigns,^55^^,^^56^^,^^59^ (6) superiority, noninferiority, or equivalency trials of new and existing tools,^34^^,^^57^ (7) incorporating new systems for targeting and increasing coverage,^33^^,^^43^^,^^57^ (8) combinations or layering of interventions,^29^ and (9) cost-effectiveness comparisons between interventions.^9^^,^^57^ For many of these questions, there is currently a lack of evidence within the existing literature to inform NMCP policy. For example, the few published studies that have examined the impact of IRS withdrawal found increases in malaria case incidence, but the extent of the increase compared with the pre-intervention period or measures to mitigate the impact of withdrawal is less well characterized.^27^^,^^30^^,^^51^^,^^55^ Similarly, there is limited evidence regarding the relationship between the level of IRS coverage and the reduction in malaria burden, with existing studies demonstrating that coverage as low as 50% reduced the risk of malaria,^60^ whereas the WHO has recommended coverage of 80% or above.^61^

#### Vector control evaluation study design considerations.

Effective use of high-quality routine data for observational analyses requires evaluators to think creatively about the natural experiments produced during intervention implementation to answer key questions as defined by policymakers. Observational studies leveraging routine data sources are typically conducted as retrospective longitudinal analyses. However, these studies can also benefit from prospective planning, particularly when considering the selection of areas that will receive each type of intervention or combination interventions and their appropriate comparison areas. Several resources already describe the various study designs available when evaluations are conducted using routine data, including before-and-after (with or without a comparison group), descriptive, interrupted time series, stepped-wedge, and dose-response studies.^22^^,^^26^^,^^62^ In some cases, evaluators may combine a CRCT design with routine data sources to provide additional information beyond what is available through the CRCT.^31^^,^^32^ The selection of the study design may vary based on the study objectives and the context in which the study is being implemented. Beyond the study design, several other factors should be considered when planning a vector control impact evaluation.

#### Evaluation period.

To account for seasonal changes in malaria case incidence, it is best to include at least 2 to 3 years of a baseline period before the intervention occurs. However, this is challenging in areas that have routinely received ITNs or IRS campaigns or where surveillance systems have experienced rapid improvements or changes in data systems and structures. If an appropriate baseline period is not available, use of a dose-response analysis can be considered if there is sufficient variation in intervention coverage. For example, a dose-response approach was used in an ITN evaluation conducted in Zambia in which no baseline period was available, as full-coverage ITN campaigns had been implemented in the country since 2005.^38^

When determining the follow-up period after the intervention, options are to use the intended period of protection, the functional period of protection, or a model that combines both. The intended period of protection can be defined based on the frequency of implementation. Insecticide-treated net mass campaigns are recommended to be repeated every 3 years.^63^ Indoor residual spraying campaigns are typically conducted once a year, and in some cases twice a year.^55^ Although using the intended period of protection represents the full period between interventions, evaluations are designed to assess the real-world effectiveness of the vector control intervention. Based on studies conducted in multiple countries, there is evidence that IRS and ITN products may last for a longer or shorter period of time than the intended period of protection. Depending on the ITN type, net use environment, and net care, one study has shown that ITN survival ranged from 1.6 to 5.3 years across four countries, with another study estimating median survival at 1.64 years across 40 countries.^64^^,^^65^ Similarly, IRS residual efficacy can range from 1 month to more than 12 months depending on the environment, wall type, and insecticide, with some newer active ingredients, such as clothianidin, lasting longer than 12 months.^66^^–^^68^ For this reason, when evaluations are conducted, it is useful to evaluate a product for the functional period of protection in addition to the intended period. Locally collected data on ITN durability and IRS residual efficacy can provide important information on the expected duration of the product and are often available through public sources, including ITN durability monitoring reports and PMI-funded entomological monitoring reports.^69^^,^^70^ Inclusion of both the intended and functional periods of protection in the analysis can help policymakers answer the more nuanced question of whether the intervention provides adequate protection until the next intervention is implemented or whether the intervention is effective but over a shorter time period and may need to be carried out more frequently. For example, an IRS impact evaluation conducted in Madagascar included an exposure variable to distinguish between the first 6 months after IRS and the 7 to 12 months since the most recent IRS campaign, reflecting the documented IRS residual efficacy.^59^ These intended and functional periods can also be used to assess the impact of discontinuing vector control activities. Two studies in Ghana and Mali compared the 6 months after IRS campaigns with the same 6 months the following year to assess the impact of IRS withdrawal.^27^^,^^30^ Follow-up periods can also be extended to assess longer-term impacts of vector control interventions when interventions are discontinued. In Uganda, evaluators assessed the impact of IRS for 31 months among health facility catchments after IRS withdrawal.^55^ The policy questions should guide the selection of the follow-up period, with evaluators clearly explaining in the resulting documentation why the follow-up period was chosen.

#### Unit of analysis (time, spatial).

Routine health systems data are often available at the health facility–month level within a national HMIS. In some instances, data may be available at the level of the community health worker (CHW) or reported weekly, such as in the community health program in Zambia or the Malaria Epidemic Early Detection System (MEEDS) system in Zanzibar or even at the individual level in areas where case investigations are conducted.^71^^,^^72^ Wherever feasible, the spatial unit of analysis should match the level at which the interventions were conducted. Many recent evaluations have used either district-month or facility-month as the unit of analysis for the study (see Supplemental Table 1). Facility-month is often preferred, as it provides a greater number of units for analysis than district-month. This can be particularly useful for IRS evaluations, when IRS is not implemented nationwide but instead in select districts. The facility-month analysis is also preferred when the exposure variable of interest (i.e., ITN or IRS coverage) is available for each health facility. The main barrier to analyzing the facility level is often the lack of reliable data on the health facility catchment population.^26^^,^^27^ As long as a suitable population data source is available, which is increasingly available with geospatial population estimates (see Defining outcome and exposure variables), the more granular data at the health facility–month or lower level provide a larger set of observations for data analysis and reduce the risk of bias or misclassification from incomplete data at lower levels.

### Defining outcome and exposure variables.

#### Outcome: Calculating malaria case incidence.

Although some vector control evaluations using routine data use other indicators of malaria burden, such as malaria hospitalization,^42^^,^^45^^,^^73^^–^^75^ death,^36^^,^^39^^,^^42^^,^^48^^,^^49^^,^^74^^,^^75^ test positivity rate,^50^ or low birth weight,^76^ the majority use malaria cases^36^^,^^39^^,^^40^^,^^54^^,^^58^^,^^74^^,^^75^^,^^77^^–^^80^ or malaria case incidence.^27^^–^^34^^,^^37^^,^^38^^,^^41^^,^^43^^,^^45^^–^^53^ With the increase in access to rapid diagnostic testing (RDT), microscopy or RDT-confirmed malaria case incidence is widely available and is typically preferred over the use of clinically diagnosed or total malaria cases (see Table 2).

**Table 2:** Outcome and exposure variables

Variable type	Indicator	Calculation	Data Source(s)	Notes/Limitations
Outcome variable	Malaria case incidence per 1,000 population	Numerator: number of confirmed malaria cases	HMIS	Multiple data elements may need to be combined. Consult with those familiar with the HMIS; check whether zeroes are stored in the system or if they are replaced with a null or missing value (can be biased by treatment-seeking behavior, RDT stock levels, and changes in community health worker scale-up over time). The preferred unit of analysis is health facility catchment if catchment population data are readily available. If not, incidence rates should then be calculated at the district level.
		Denominator: population at risk	Government census, local head counts, geospatial data sources matched to health facility coordinates	Geospatial sources are usually the most complete at the health facility catchment level; population data sources should be triangulated for verification.
Exposure variables	Intervention status	Binary variable (yes/no)	Campaign or program documentation	Variable should be collected and calculated at the same unit of analysis as the outcome indicator (i.e., district or health facility level).
	Intervention coverage: proportion of the population at risk potentially protected by the intervention	Numerator: number of people potentially protected by the intervention ITNs: number of ITNs distributed multiplied by 2 IRS: number of people living in structures sprayed with IRS	Campaign or program documentation	Coverage rates are useful in combination with binary intervention status variables to assess the impact of higher coverage rates and for dose-response analyses when there is no suitable comparison group.
		Denominator: population at risk	Government census, local head counts, geospatial data sources matched to health facility coordinates	Population data should be from the same source used to calculate the outcome variable.

HMIS = health management information system; IRS = indoor residual spraying; ITN = insecticide-treated net; RDT = rapid diagnostic testing.

#### Malaria cases.

Confirmed malaria case incidence, normally defined as cases per 1,000 population, is the most common outcome indicator used in vector control evaluations that use routine data. There are often subjective decisions to be made about which variables within a national HMIS should be used to determine this indicator. In some cases, there is a confirmed malaria cases data element, as well as data elements for the number of positive RDT results and microscopy tests. Data may also be stratified by age group, and evaluators must determine whether to include individual age groups or combine indicators into an all-ages analysis. A national HMIS data dictionary or other tools that support data systems exploration can be useful to understand the data available.^72^^,^^81^ Evaluators who are not NMCP staff members or highly familiar with the data systems should identify a contact person who is involved with the ongoing management of the system who can support the interpretation of key indicators, particularly considering any changes in variable definitions or data collection processes over time.

Many HMIS systems store zero values as missing data as a space-saving measure for national servers. This practice makes it difficult to distinguish between true zero values and missing data for malaria cases and other indicators. In high burden areas, null values are more likely to be missing data. However, in low burden areas, zeros are more likely to be true values and could constitute a larger portion of the dataset. When data completeness is low (see Table 3), sensitivity analyses could be done, treating the values as missing and then as true zeros to test the robustness of the findings.

**Table 3:** Covariates and contextual factors

Indicator	Definition	Data Source	Purpose
HMIS*			
Testing rates among febrile patients	% of febrile patients who tested for malaria (number of patients tested/Number of febrile patients)	HMIS	To account for fluctuations in parasitological diagnosis
Non-malaria outpatient attendance	Total outpatient attendance minus confirmed malaria cases	HMIS	To control for variations in treatment-seeking behavior
IPTp	% of women receiving full course of IPTp per national guidelines	HMIS	To control for other malaria prevention measures
Continuous ITN distribution	% of pregnant women and infants receiving ITNs during health facility visits	HMIS	To control for other malaria prevention measures
RDT stockout	Varies by country but is often (1) number of days with an RDT stockout, (2) RDT stockout that lasted for > 7 days (yes/no), or (3) any RDT stockout (yes/no)	HMIS or LMIS	To control for periods where RDT stockouts can impact the number of confirmed cases
Data completeness	% of months during the study period with data available	HMIS	To determine how missing or inconsistent data may skew the results
Climate and ecology†			
Precipitation/rainfall	Monthly averages calculated in millimeters; values should be lagged 1–3 months	Climate Hazards Group InfraRed Precipitation with Station Data; local weather stations	Climate variables are often positively correlated with malaria cases and should be considered for inclusion as a covariate in the evaluation model.
Vegetation	Index values calculated as monthly averages; values should be lagged 1–3 months	Famine Early Warning Systems Network; MODIS Vegetation Index Products (NDVI and EVI)	
Temperature	Monthly averages calculated as °C; values should be lagged 1–3 months	MODIS Land Surface Temperature and Emissivity; local weather stations	
Elevation	Static value calculated in meters	U.S. Geological Survey’s Terra Advanced Spaceborne Thermal Emission and Reflection Radiometer; Shutter Radar Topography Mission	
Routine survey data‡			
Treatment-seeking behavior^81^	Proportion of children under 5 years old with fever in the last 2 weeks for whom advice or treatment was sought	DHS, MIS	To adjust for differences in treatment-seeking behavior by region
Private sector treatment^3^	Proportion of children under 5 years old with fever in the last 2 weeks for whom advice or treatment was sought from the private health sector	DHS, MIS	To adjust for private treatment seeking, which is often not accounted for within HMIS data
Population ITN access^82^	Total number of individuals who could sleep under an ITN if each ITN in the household is used by two people/Total number of individuals who spent the previous night in surveyed households	DHS, MIS; ITN durability monitoring surveys; malaria behavioral surveys	To adjust for population access to ITNs per national surveys; can be used in collaboration with or instead of ITN program distribution data
ITN use^81^	Number of individuals who slept under an ITN the previous night/Total number of individuals who spent the previous night in surveyed households	DHS, MIS; ITN durability monitoring surveys; malaria behavioral surveys	To explain why ITNs may or may not have been effective by determining how often they are used and where
ITN survivorship^83^	Proportion of distributed nets still available for use as intended in the households to which they were given after a defined period	ITN durability monitoring surveys	To explain why ITNs may or may not have been effective by determining whether ITNs distributed during the previous campaign are still available
Entomological surveillance data§			
Human biting rate^84^	Number of adult female vectors collected divided by the total number of collector nights (disaggregated by indoor/outdoor collection)	Entomological surveillance	To assess the impact of vector control interventions
Vector density^84^	Total number of vectors collected resting inside per house per day		To assess the impact of vector control interventions
Indoor resting density^84^	Total number of vectors collected resting inside per house per day		To assess the impact of vector control interventions
Sporozoite rate^84^	Number of adult female vectors positive for CSP divided by the total number of adult female vectors tested for CSP		To assess the impact of vector control interventions
Entomological inoculation rate^84^	Number of bites per person per unit time multiplied by sporozoite rate		To assess the impact of vector control interventions
IRS residual efficacy	Number of adult female *Anopheles* malaria vectors that die after exposure to insecticide on treated surface in the field divided by the total number of adult female *Anopheles* malaria vectors exposed to treated surface Number of months for which insecticide residual efficacy remains above 80%		To explain the duration of IRS efficacy
Resistance frequency^84^	Proportion of adult female vectors alive after exposure to insecticide 100% – (number of dead or incapacitated *Anopheles* malaria vectors/Total number exposed to a discriminating concentration of insecticide in standard bioassays)		To explain why interventions may or may not have been effective by determining whether local mosquito populations are susceptible to the insecticide used
Resistance status^84^	Classification of adult female vector populations as confirmed resistant (< 90%), possibly resistant (90–97%), or susceptible (≥ 98%)		To explain why interventions may or may not have been effective by determining whether local mosquito populations are susceptible to the insecticide used
Resistance intensity^84^	Classification based on proportion of mosquitoes dead or incapacitated after exposure to 5× and 10× intensity concentrations of an insecticide in a standard bioassay, whereby < 98% after 10× exposure = high-intensity resistance; ≥ 98% after 10× exposure but < 98% after 5× exposure = moderate intensity resistance; ≥ 98% after 10× and 5× exposure but < 98% after 1× exposure = low-intensity resistance		To explain why interventions may or may not have been effective by determining whether local mosquito populations are susceptible to the insecticide used
Biting time^84^	Number of adult female vectors that attempt to feed or are freshly blood-fed, per person per unit time, usually expressed per 2-hour increments		To explain why ITNs may or may not have been effective by determining whether individuals are likely to be asleep and under ITNs; newer studies compare biting time with human behavior studies to determine when individuals are indoors and asleep
Biting location^84^	Proportion of attempted bites or successful blood-feeds by adult female vectors indoors and outdoors, per unit time		To explain why interventions may or may not have been effective by determining how often mosquitoes bite indoors, where IRS and ITNs are effective
Population ITN access^82^	Total number of individuals who could sleep under an ITN if each ITN in the household is used by two people/Total number of individuals who spent the previous night in surveyed households		To adjust for population access to ITNs per national surveys; can be used in collaboration with or instead of ITN program distribution data
ITN use^81^	Number of individuals who slept under an ITN the previous night/Total number of individuals who spent the previous night in surveyed households		To explain why ITNs may or may not have been effective by determining how often they are used and where

CSP = circumsporozoite protein; DHS = demographic and health survey; EVI = enhanced vegetation index; HMIS = health management information system; IRS = indoor residual spraying; ITN = insecticide-treated net; IPTp = intermittent preventive therapy in pregnancy; LMIS = logistics management information system; MIS = Malaria Indicator Survey; NDVI = normalized difference vegetation index; RDT = rapid diagnostic testing.

* A designated point of contact can help to determine the correct indicator and source for RDT stockout data. Data completeness should be used as a covariate in the model when there is a wide variation across the unit of analysis.

† Data should be collected at the same unit of analysis as the outcome and exposure variables.

‡ Data on treatment-seeking behavior are often available only at the regional or provincial level and will often be used contextually. An identified point of contact can help to determine if data from the private sector are included in the HMIS or not.

§ All of the entomological indicators below can be considered for both ITN and IRS evaluations, with the exception of residual efficacy, which is for IRS only.

Another common challenge in calculating malaria cases for an evaluation occurs when CHWs contribute case data to the HMIS. Community-based malaria case management typically expands gradually over time, and with that expansion, malaria case counts often increase. If CHW data are stored within the HMIS and disaggregated from health facility reports, the percentage of cases seen by CHWs could be used as a covariate to control for the potential increase in diagnoses where CHWs are more active.^73^^,^^85^ In countries or subnational areas where CHWs are active but case data are not stored or disaggregated, evaluators may have difficulty controlling for the effects of CHW activity on case counts, which could lead to bias in the evaluation results.

#### Population.

Many HMIS systems include population data through extrapolations from census data, local head counts, or both. Census data are the official data source for population statistics in most countries and are usually available for use by national programs through national statistics offices. However, longer time periods between the original census data collection and the current evaluation year increase the potential risk for data inaccuracy, including geographical boundaries that no longer match current administrative structures and population estimates that are often lower than those observed through other sources. For example, vector control interventions using census projections as the areal denominator to estimate the proportion of the population protected often find coverage rates over 100%, as do other mass campaigns such as seasonal malaria chemoprevention and immunization programs.^86^

For local head counts, the MOH or NMCP often asks local governments to provide a count of the populations in their geographic area either on an annual basis or when countries are preparing for mass campaigns, such as ITN or IRS campaigns. Local governments may use multiple methods to obtain these estimates, including receiving counts from village or area chiefs, using results from a recent enumeration for another intervention (e.g., a recent public health intervention or research project that required household registration), or conducting a local census or ad hoc registration process for the campaigns (which may include primary data collection, a projection based upon national census, or other estimate methods). The methods used to produce these estimates are not often documented, may not cover all the evaluation areas, and may vary by local governments, making it difficult to compare estimates from one area with those from another. This method of estimation is also vulnerable to over- and underestimation. For example, in a study of mass drug administration in an urban area of Liberia, a Médecins Sans Frontières census was used in collaboration with estimates from community leaders to initially project a population of 300,000. The population was later verified as 551,971 by distribution teams during the first round and corrected to 558,483 in the second round—a 186% increase from the initial projection.^87^ Local head counts are usually a trusted source of information, particularly when the census is outdated and may not reflect recent population movements or changes.

Many HMIS systems have census extrapolations or local head counts available at the district level. Although health facility catchment populations within an HMIS are increasingly available, they are still not available or complete in many countries. Where they are available, these sources may be preferred by NMCPs as the official population sources. However, the process for deriving facility-level head count estimates may differ between localities and is often not well documented. In addition, if facility-level head counts are not systematically and regularly updated, it may be difficult or impossible to determine how the opening or closing of facilities may impact population estimates of neighboring facilities.

Where health facility catchment populations are not available or complete within an HMIS, populations can be estimated using a variety of well-documented geospatial techniques.^26^^,^^86^^,^^88^^,^^89^ These techniques use publicly available gridded population estimates to assign a population to a health facility catchment based on its geospatial coordinates. Maintenance of complete facility geo-registries is necessary for geospatial-based methods. However, unlike head counts, facility locations are static and do not depend on variable estimation protocols. Geospatially derived population estimates can be readily updated and designed to consider only active facilities. Triangulation of population data sources and review by national stakeholders can help to determine the best population option to use and can build confidence in the evaluation results. Where civil registration and vital statistics systems are actively maintained, they can also be used to support the calculation and revision of population estimates. Although these systems are currently weak in many malaria-endemic countries, this could be a useful source in future evaluations as these systems improve.^90^

#### Exposure: Calculating ITN and IRS coverage.

Common methods of calculating ITN and IRS coverage for evaluations include binary variables that indicate whether an area received an intervention, a continuous variable that measures the proportion of the population that is expected to be protected by the intervention, or a more complex model that attempts to capture both initial population coverage and the estimated effective duration of the intervention over time (see Table 2).

Binary intervention implementation variables are the simplest measures to collect and report, and they are less subject to biases in coverage estimations. As noted under the unit of analysis, ideally, the data on whether a given area received an intervention will match the lowest level of data available for the outcome variable. For example, if the intervention was implemented and data are available at the village level, then it would be ideal to have village-level case data available. Similarly, if health facility catchment is the lowest level of case data, then it is preferable to have data on whether each health facility received the intervention. A main challenge in using the health facility level or lower as the unit of analysis is the ability to match the spatial units between the intervention and case data. As intervention data are often stored outside of an HMIS, the spatial unit names or hierarchies may not be the same as those used in the HMIS. If vector control implementation data are not available at the same level as the outcome variable (i.e., only at the district level and not at the heath facility level) or coverage of the intervention is not uniform, with areas receiving higher or lower coverage of the intervention levels, the impact of the intervention may be misspecified.

Population coverage can be defined as the proportion of the population at risk that is protected by the intervention.^86^ A commonly used indicator for assessing IRS coverage is the proportion of households or structures sprayed out of the total targeted or found.^86^ Although such indicators are useful for program operations and monitoring, they cannot easily be extrapolated to the population for use in impact evaluations.^86^ Ideally, IRS population coverage would be calculated as the number of individuals residing in sprayed households divided by the estimated population at risk or the population in the spatial area of interest.^77^^,^^91^ However, this is feasible only if the population protected is recorded during IRS interventions and population estimates are available. The ITN population distribution coverage can be calculated as the number of ITNs distributed multiplied by 2 and divided by the estimated population, based on the WHO recommendation that one ITN should be distributed for every two people (i.e., universal coverage).^64^ It may also be multiplied by 1.8 to account for households with an uneven number of members.^64^^,^^66^ The accuracy of both IRS and ITN coverage rates relies on the quality of the program intervention data collected and, as with malaria case incidence, the selection of the population data source. Population coverage estimates are useful for dose-response analyses when there is not a suitable comparison area and there is adequate coverage variation to detect a difference.^38^ Similar to binary estimates, coverage that is available at the unit of analysis (i.e., health facility or district) is preferred. In many cases, evaluators will use the binary variable as the exposure variable within the statistical model and use continuous coverage variables to provide additional context for interpretation.^30^^,^^33^^,^^43^^,^^47^^,^^52^^,^^74^

Newer statistical models of estimating coverage are incorporating multiple factors to better estimate the duration and use of the interventions. One study in Madagascar, which assessed the impact of IRS on malaria case incidence, incorporated the IRS status at the health facility level (binary), time since the IRS intervention (0–6 months versus 7–12 months based on insecticide residual efficacy data), number of past consecutive years of IRS, and number of structures sprayed/number of structures found.^59^ The study also controlled for the impact of ITNs using ITN survivorship from an ITN durability monitoring study to project ITN survivorship across the study areas.

In addition, modelers have produced global maps of ITN and IRS coverage for sub-Saharan Africa by blending multiple datasets.^65^^,^^91^ These studies integrated program data, demographic health survey data, published articles, WHO World Malaria Reports, and data from ITN manufacturers to produce granular datasets to calculate estimates of intervention coverage from the national level to the 5 by 5–km pixel.

Finally, some areas that receive IRS and ITN interventions will be closer to the border of the intervention than others. Health facilities or CHWs in border areas are more likely to include patients from areas that did not receive the vector control intervention being studied. In these situations, sensitivity analyses can be conducted including and excluding prespecified border facilities to test the robustness of the findings. For example, in an ongoing analysis in Sierra Leone, sensitivity analyses will also be conducted excluding health facilities where <80% of the catchment population, as determined through geospatial modeling, is located within the intervention or comparison district. If individual-level data are collected, with the village of origin for each resident recorded and matched to the intervention areas, the primary analysis can be restricted to only those patients who originate in areas that received the intervention. However, this level of detail is often not available without collecting data directly from health facility registers that contain village level information.^32^

### Identifying covariates and contextual factors.

It is important to be able to account for potential biases in the exposure, outcome, or impact estimates. Ashton et al.^26^ provide a detailed list of such factors to consider, including access to health care and treatment seeking for malaria, malaria diagnostic practices, and climate data. For vector control evaluations, there are a variety of routine data sources and indicators that can be useful in the analysis and interpretation of results (see Table 3). These factors can be broadly divided into two groups, covariates that are included directly within the statistical analysis and contextual factors, which are descriptive summaries of key data used to describe the evaluation context and support the explanation of key findings. Whether to include a variable as a covariate or contextual factor can be determined by the availability of the data (are they available at the unit of analysis?) and generalizability (if they are not available at the unit of analysis, can they feasibly be extrapolated and applied?). Given the many covariates and contextual factors that should be considered in vector control evaluations, the triangulation and interpretation of these factors can be complex. However, it is important to review these data sources and consider, in collaboration with key stakeholders, their potential impact on the intended outcome.

#### Other health service delivery data.

Beyond malaria cases and population, health service delivery data often contain additional data elements that are useful either as covariates in the model or as contextual variables. Total outpatient visits can be used to calculate non-malaria outpatient visits to adjust for differences in treatment-seeking between health facilities and over time. Malaria testing rates (proportion of malaria suspects receiving a diagnostic test) or RDT stock data, available within the HMIS or a separate logistics management information system, should also be incorporated to adjust for differences in access to diagnostic testing, which could potentially bias the number of confirmed malaria cases. The HMIS also often contains data on other malaria prevention interventions, including intermittent preventive therapy in pregnancy and continuous distribution of ITNs through antenatal care and the expanded program on immunization.

#### Entomological surveillance data.

Entomological surveillance data, such as that routinely collected by national programs or by research or implementing partners, can be useful to explain trends in malaria case incidence.^78^ Where data are available for the entire evaluation period and across both intervention and comparison areas (if any), vector density, human biting rate, indoor resting density (for IRS), sporozoite rate, and entomological inoculation rate (EIR) can be used to assess the impact of vector control interventions on vector populations.^92^ Although the sporozoite rate and EIR are malaria transmission measures (before post-inoculation individual factors such as immunity, age, nutrition, and genetics and systems factors such as access to treatment), they require more complex analyses, can be difficult to measure in low-transmission settings, and have limited generalizability beyond the sites in which they were collected.^82^^,^^92^

Other entomological indicators are as follows: Resistance frequency and intensity can be useful in determining whether an insecticide is still effective against mosquitoes in the areas of interest. Biting time and location can be useful in determining where and when humans are likely to be protected by ITN and IRS interventions, namely indoors and at night, as well as potential gaps in coverage.^83^^,^^84^ Entomological surveys may also contain information on ITN availability and use among the few households visited for entomological collections.^83^ This varies from collecting the type and number of ITNs and the proportion of household members who slept under an ITN to collecting hourly information on human behavior to quantify when individuals are indoors and under ITNs.^34^^,^^83^ Although surveys of these factors may not necessarily capture the full spectrum and extent of human behaviors in relation to vector control interventions owing to their limited sampling, they may still be useful in partially explaining HMIS data trends.

#### Routine surveys.

Nationally representative surveys or vector control monitoring and evaluation surveys can be another useful source of contextual information. National household surveys, such as demographic and health surveys, are often ill-suited for evaluations of specific campaigns, as they are usually collected every 3 to 5 years during time periods that may not align with the evaluation and data are often available only at the provincial or regional level.^86^ As IRS is often implemented at the subregional level, the most recent guidance for household surveys no longer recommends including questions related to IRS.^93^ However, these surveys can be useful in calculating coverage and use indicators for ITNs and understanding care-seeking behavior, and they can provide useful contextual information on how these factors may differ by region.

Insecticide-treated net durability monitoring surveys are typically conducted on an annual basis up to 3 years after an ITN campaign, with the primary purpose of evaluating the bioefficacy and physical integrity of ITNs.^64^^,^^69^ If conducted within designated areas for vector control evaluation, these small-scale surveys can also be a useful source of information on ITN survivorship, attrition, and use. Sampling for ITN durability monitoring is based on a cohort of ITNs and is not representative of the human population. Malaria behavioral surveys aim to understand drivers of behavior and provide key information on ITN use, care, and repair; care seeking for malaria testing and treatment; and IRS acceptance.^94^ These surveys collect data on human behavior but are restricted to select areas within a given country. Despite the subnational sampling methods, ITN durability monitoring and malaria behavioral surveys can still be useful in supporting the interpretation of key findings.

#### Climate and ecology.

Key climate and ecological variables include precipitation (rainfall), vegetation indices, temperature, and elevation. Climate variables are available from modeled geospatial data, which are publicly available for use from sources such as the Climate Hazards Group InfraRed Precipitation with Station Data for rainfall^88^; enhanced vegetation index for vegetation^95^; land surface temperature and emissivity for temperature^96^; and the Global Multi-resolution Terrain Elevation Data for elevation.^97^ These variables can also be accessed from national meteorological departments and local weather station data.^51^ Climate and ecology variables should be calculated at the same unit of analysis as the outcome and exposure variables, especially when climate and ecology variables are used as covariates in the model. Similar to geospatial population data, health facility coordinates are used to match climate and ecology data to health facility catchment areas. Climate and ecology variables are calculated as total,^39^ mean values,^58^^,^^74^ or anomalies, or they use a mix of approaches.^33^^,^^37^ It is also considered best practice to incorporate climate variables as lagged values, given that changes in climate may not impact changes in malaria cases until 1 to 3 months later.^26^

#### Other programmatic data.

In the analysis, NMCPs and evaluators should include other programs or interventions relevant to the evaluation areas. This could include other malaria interventions such as seasonal malaria chemoprevention^29^ or mass drug administration, mass screening, or other health programs. Data for these can often be obtained from the relevant national health programs. For other events that may impact the intervention or data sources—such as health worker strikes, political instability and insecurity, or population migration—detailed data may not be available. Public news sources can often provide broad information and context for the analysis.

### Potential data quality challenges and how to address them.

Although the availability and quality of routine health systems data continue to improve, data quality is still a noted limitation that could lead to biased results for vector control evaluations. When conducting evaluations using routine data, evaluators should characterize the quality of the data.

Increasingly, NMCPs and implementing partners are conducting routine data quality assessments in which timeliness, completeness, accuracy, consistency, and validity are assessed through review and verification of data within health facilities and the HMIS. Where recent data quality assessments or audits are available, these results can provide important contextual information to inform the interpretation and use of findings. Before or as part of an evaluation, evaluators may also conduct data quality assessments within evaluation areas. Where tools are not already available from national sources, global tools can be adapted to support data quality assessment efforts.^99^^,^^100^ In some cases where initial data quality assessments reveal gaps in data from HMIS sources, evaluators may recommend collecting routine data directly from health facility registers. Finally, when on-the-ground data quality assessments are not possible, remote data quality checks in which HMIS data are reviewed for key data quality indicators can still be useful in assessing data quality before an evaluation is conducted. Data quality checks on all outcome, exposure, covariate, and contextual variables should be incorporated into any evaluation and steps taken to mitigate their impact. Plotting raw data can help to identify erroneous values and outliers, and clear rules can be documented in a data cleaning protocol to flag data points that fall outside of the pre-identified criteria, which can be reviewed for potential exclusion. Data completeness can also be assessed by calculating the proportion of observations with missing data. Health facilities or other observational units with missing data can be removed from the analysis if they do not meet a data completeness threshold.^33^^,^^52^^,^^73^ For example, an evaluation of standard and PBO ITNs on pregnancy outcomes in Uganda removed facilities that did not have data for at least 25 months of the 38-month evaluation period or when covariates were systematically missing.^76^ Alternatively, imputation methods can be applied to the missing data.^43^^,^^51^ For example, an evaluation of scaling up anti-malaria interventions in Ghana found that data for 10% of the months were missing. The researchers then imputed data for missing months by taking the average of the same month from the other years of the evaluation period.^74^ Finally, data from all health facilities can be included in the evaluation, and reporting completeness rates can be integrated as a covariate in the evaluation.^37^

### Using evaluation findings to inform national policy, strategy, and program implementation.

Evaluations using routine data are intended to inform national malaria policies, strategies, and implementation. Without this crucial step, the evaluation becomes an academic exercise, with little practical use. There are several steps that evaluation teams can take to increase the potential for evaluation findings that can inform national decision-making. First, evaluators should define and engage relevant stakeholders and decision-makers early in the evaluation process. As noted under study objectives above, vector control and NMCP leadership should identify the specific policy decisions that the evaluation will inform. Evaluators can facilitate a discussion of evaluation use by asking stakeholders three questions: (1) How do you anticipate the results of the evaluation will be used? (2) Who is interested in the outcome(s) of this evaluation? (3) What do you want to do differently as a result of engaging with the evaluation findings? In some cases, it may also be helpful to facilitate a thought experiment with decision-makers exploring potential outcomes of the evaluation and how they might use the results in each case. For example, when the impact of standard pyrethroid and new types of ITNs is being compared, what decision might the NMCP make if the results show that the new ITNs performed better or if the evaluation results show no difference in impact?

Evaluators should also consult decision-makers on what type of evaluation product—a report, presentation, evidence briefs, peer reviewed publication, or other product—they would prefer to inform their decision and how and where these products should be presented. Previous research has demonstrated that short, solution-oriented take-home messages, such as evidence briefs, tend to be more effective than research reports.^98^ Finally, evaluators should map out key decision points, such as malaria strategic planning processes, midterm strategy reviews, and donor cycles such as Global Fund applications or PMI malaria operational plans. Evaluators should set expectations with decision-makers regarding when interim or final results will be available to inform these key strategy points and determine what specific content decision-makers would like to include.

During the evaluation process, evaluators can engage key stakeholders and decision-makers in reviewing initial descriptive findings and any interim results. This early exploration can help to refine variable definitions and suggest practical solutions to data quality challenges, ensuring that the final product meets NMCP needs. Finally, when the evaluation is complete, the results should be presented in a way that is easily interpretable and usable by program staff. For example, rather than presenting risk ratios, results can be presented in terms of cases averted per population or a percent change in malaria case incidence, comparing intervention and comparison areas where feasible. It may also be helpful to describe whether the intervention resulted in changes in stratification level (i.e., from high to medium burden) according to national or WHO stratification guidelines.

## CONCLUSION

Although CRCTs provide the highest quality of evidence for impact, they are costly. Well-designed observational, quasi-experimental evaluations using routine epidemiological, entomological, and programmatic data sources can allow NMCPs to gain important insights into the impact of vector control interventions at a lower cost, at a more granular level, and with greater breadth across the country than commonly available through RCTs or other primary data collection methods.

This article provides methodological recommendations to support MOHs and NMCPs in conducting strong vector control evaluations using routine data that lead to evidence-based policy development and program implementation. Evaluations should aim to address the most relevant policy questions, going beyond the impact of vector control on malaria case burden to address questions such as optimal timing, duration, dosage, combinations of interventions, and the intended and functional periods of protection.

Key recommendations for designing vector control evaluations and selecting variables include (1) using the health facility catchment population month or lower as the unit of analysis, when that level of granular data is available, (2) using binary measures of intervention coverage and variables that incorporate the duration and use of the intervention as exposure variables, and (3) incorporating both covariates and contextual factors, including entomological data, to describe the evaluation context and support the explanation of key findings.

Key recommendations for the process of implementing vector control evaluations include (1) ensuring an in-depth understanding of the data systems being used, including changes in systems and data or variable structure over time, (2) assessing data quality through either facility-based data quality audits or remote data quality checks and taking steps to reduce bias in the data, such as removal of incomplete data for health facilities or imputation, and (3) triangulating population data sources to determine the best population option to use and build stakeholder confidence in the evaluation results.

Finally, key recommendations for ensuring that evaluation results are used include engaging stakeholders to define specific (1) evaluation objectives and use cases for evaluation findings, (2) evaluation products that would be useful for decision-makers, and (3) key decision points and their timing to ensure that evaluation results are incorporated into the national malaria policy, strategy, and program implementation.

Continued strengthening of malaria surveillance and vector control data systems, data integration, including publicly available sources of climate and population data, and the increasing focus on data-driven vector control decision-making will strengthen the design and implementation of vector control evaluations and ensure their use in guiding NMCP policy and strategy. The increased availability of routine data also reduces the cost of conducting these observational impact evaluations, making them more within reach for NMCPs seeking an evidence-based policy to support national vector control decisions. Future evaluations should continue to build upon this guidance, further identifying methods and considerations that lead to strong vector control evaluations and data use.

## Supplemental Materials

10.4269/ajtmh.22-0757 Supplemental Materials
