# Supplementary material for: Process and Methodological Considerations for Observational Analyses of Vector Control Interventions in Sub-Saharan Africa Using Routine Malaria Data
Source: Am J Trop Med Hyg. 2023 Aug 21;112(1 Suppl):17–34. doi: 10.4269/ajtmh.22-0757 (PMC11720682; doi:10.4269/ajtmh.22-0757)
Supplement: Supplemental Materials [file tpmd220757.SD1.pdf]

Table S1. Literature review of vector control impact evaluations using routine HMIS data

Article	Country	Evaluation Question(s)	Time Period	Design & Analysis	Unit of Analysis	Outcome	Exposure	Covariates	Contextual Variables	Data Quality Checks
Abong'o et al. 2020[75]	Kenya	(1) What is the impact of IRS on entomological indices? (2) What is the impact of IRS on epidemiological indices?	26 months pre- and 15 months post-campaign	Pre/post comparison using an ARIMA model used to detect changes in malaria cases.	Health facility; 5 facilities in 1 county	Malaria cases	IRS status (binary)	None reported	Vector density and species, insecticide resistance, sporozoite rate, biting rates, residual efficacy	None reported.
Aregawi et al. 2017[72]	Ghana	What is the impact of scaled up use of ACTs and ITNs on malaria cases, admissions, and deaths?	6 years pre- and 5 years post-campaign	Interrupted time series using segmented log-linear regression.	Hospital; 88 hospitals in 3 epidemiologic zones	Malaria cases and deaths stratified by <5 and ≥5 years	Scale up period (binary)	Non-malaria outpatient consultations, non-malaria admissions, non-malaria deaths, patients w/ and w/o health insurance, rainfall, temperature	ITN coverage, IRS coverage, ACT availability, trends in child mortality	Missing data was imputed (10% of all months missing).
Ashton et al. 2019[32]	Zanzibar (Tanzania)	What is the impact of ACTs, IRS, and ITNs on monthly malaria incidence rates?	44 months pre-interventions, 28 months post-ACT only; 10 years post-ACT plus vector control	Interrupted time series using a random-effect negative binomial regression model.	District; 8 of the 10 districts in Zanzibar	Confirmed malaria case incidence per 100,000	Intervention status (no intervention, ACT-only, ACT plus vector control)	Rainfall, vegetation, temperature, # of facilities reporting, total all-cause outpatient attendance, % of OPD attendees receiving malaria test	None reported; all used as covariates in the model	Reporting included as covariate. High data completeness reported. Districts removed that did not have data on parasitological diagnosis in pre-intervention period.

Article	Country	Evaluation Question(s)	Time Period	Design & Analysis	Unit of Analysis	Outcome	Exposure	Covariates	Contextual Variables	Data Quality Checks
Kanyangarara et al. 2016[36]	Zimbabwe	What is the impact of switching from a pyrethroid IRS to Actellic IRS on malaria incidence?	105 weeks pre- and 24 weeks post-intervention	Pre/post comparison using a negative binomial regression model with panel data with control group. GEE approach used to account for repeated measures correlation. RMSE to evaluate agreement between observed and model predicted cases.	Facility; 42 health facilities in 33 wards (20 malaria-prone wards selected for spraying)	Confirmed malaria case incidence per 1,000 population (weekly)	IRS status (binary)	Year, month, elevation, rainfall (6 week lag), daytime temperature (1 week lag), nighttime temperature (10 week lag), vegetation	Climate variables also used to estimate correlation between climate and incidence	One facility excluded that did not start reporting until later in the study period.
Larsen et al. 2020[38]	Zambia	What is the impact of different IRS campaign strategies on malaria incidence?	~46 months pre- and ~5–7 months post-campaign	Difference-in-differences comparison using a generalized linear model using a negative binomial regression. A similar model used to model the number of mosquitoes found at each household.	Facility; 158 facilities in 6 districts	Confirmed malaria incidence	IRS status (binary); planning strategy (health-facility targeting, geographic concentration, ecological-targeting)	Health facility type, # of tests performed, vegetation (lagged 1 month), precipitation (lagged 1 month), nighttime lights, elevation, confirmed cases (lagged 1 month)	Vector abundance, IRS coverage, ITN coverage	Additional cleaning done for entomologic indicators. Missing vegetation data imputed. For facilities with missing coordinates, district mean applied.
Machini et al. 2016[98]	Kenya	What are the trends in three key malaria indicators following the ITN campaign?	5 years covering 2 campaigns	Descriptive analysis of indicators over time.	3 epidemiological zones	OPD malaria case prevalence, % of confirmed malaria cases by age group, % of population protected by ITNs	None, trends over time	N/A	ITN coverage	None reported.
Mumbengegwi et al. 2018[42]	Namibia	Is there a correlation between malaria incidence and IRS coverage?	8 months post-campaign	Descriptive analysis of indicators. A Pearson's product-moment correlation coefficient was used to determine the strength of the relationship between incidence and reported IRS coverage.	Facility; 11 facilities in 1 region	Malaria incidence per 1,000 population	None, post-only analysis	N/A	IRS spray coverage (both administrative and reported by household), ITN distribution, additional IRS activities, active case detection activities	None reported.

Article	Country	Evaluation Question(s)	Time Period	Design & Analysis	Unit of Analysis	Outcome	Exposure	Covariates	Contextual Variables	Data Quality Checks
Oguttu et al. 2017[45]	Uganda	How do ITNs and IRS impact malaria incidence, test positivity rates, and malaria OPD attendance?	17 months pre- and 2 years post-ITN; 11 months pre-first IRS, 5 months post-first IRS, 5 months post-second IRS	Pre/post comparison using Chi square for trends to analyze annual change. Logistic regression used to estimate the monthly reduction in incidence.	District; 61 facilities in 1 district	Malaria case incidence per 1,000 population, test positivity rate, % of OPD visits for malaria	IRS status (binary)	None reported	None reported	Data from 2012 removed since HMIS was not rolled out until mid-2012. Trends in reporting rate to HMIS provided.
Okullo et al. 2017[46]	Uganda	(1) What is the impact of IRS on malaria case incidence on those <5 years?<br>(2) What is the impact of IRS withdrawal on malaria case incidence on those <5 years?	4–5 months pre- and 5–7 months post-campaign depending on year	Pre/post comparison using a Poisson regression to test the significance of incidence rate ratios. Control group was those districts that did not receive IRS.	District; 20 districts in Northern Uganda (10 IRS districts, 10 non-IRS districts)	Malaria case incidence (confirmed or clinical) <5 years per 1,000 population	Intervention status (IRS ended Nov 2014, IRS ended Jun 2015, no IRS)	None reported	Rainfall, HMIS reporting rates, ITN coverage and use, passive malaria and surveillance and behavioral change communication	Adjustment done for months with <100% reporting. Smoothing and interpolation methods also used for missing data.
Roh et al. 2022[59]	Uganda	(1) What is the impact of ITNs on pregnancy outcomes?<br>(2) What are the differences between PBO and non-PBO ITNs on pregnancy outcomes?	29 months pre- and 9 months post-campaign	Primary analysis used interrupted time series with Poisson regression to estimate campaign effects. Difference-in-difference using Poisson regression used to compare conventional and PBO ITNs.	Health facility; 21 facilities in 12 health subdistricts (HSDs)	Low birth-weight incidence; stillbirth incidence	ITN status, post-ITN period (broken down into 3-month periods to approximate pregnancy trimesters), net type (conventional or PBO)	Months since study start, months since ITN campaign, calendar and health facility fixed effects, mean maternal age at delivery, % of primigravidae, and % of HIV+ positive women	# of deliveries	Health facilities were excluded if missing at least 25 months during the study period or covariates or outcomes were systematically missing. 8 of the 32 facilities were excluded.
Topazian et al. 2021[47]	Malawi	(1) What is the impact of a mass bed net distribution campaign on confirmed malaria case incidence?<br>(2) Are PBO ITNs more effective than pyrethroid ITNs?<br>(3) Does use of IRS sustain protection relative to pyrethroid ITNs?	~8–11 months pre- and 18–21 months post-campaign depending on district	Observational, longitudinal surveillance study using Quasi-Poisson regression to estimate and compare risk.	Health facility; 711 facilities	Confirmed malaria case incidence per 100 population	Intervention status (standard ITNs, PBO ITNs, or IRS)	None reported	IRS coverage	Excluded facilities that were missing geocoordinates and 2018 data.
